# Prevalence of *Arcobacter* Species among Humans, Belgium, 2008–2013

**DOI:** 10.3201/eid2010.140433

**Published:** 2014-10

**Authors:** Anne-Marie Van den Abeele, Dirk Vogelaers, Johan Van Hende, Kurt Houf

**Affiliations:** Sint-Lucas Hospital, Ghent, Belgium (A.-M. Van den Abeele); University Hospital, Ghent (D. Vogelaers);; Ghent University, Merelbeke, Belgium (J. Van Hende, K. Houf)

**Keywords:** Bacteria, *Arcobacter*, *Campylobacteraceae*, *Campylobacter*, gastroenteritis, enteritis, colitis, septicemia, aerotolerant, diarrhea, zoonoses, food safety, Belgium

## Abstract

We examined fecal samples from 6,774 patients with enteritis in Belgium, 2008–2013. Members of the genus *Arcobacter* were the fourth most common pathogen group isolated, and the isolation rate was higher than previously reported. Culturing *Arcobacter* in a microbiology laboratory is feasible and should thus be tested for in cases of diarrheal disease.

Campylobacteriosis is the most frequently reported zoonosis in industrialized countries with an increasing incidence during 2007–2011 (*1*). In this study, bacteria of the *Arcobacter* genus, which is closely related to the *Campylobacter* genus, comprised the fourth most common pathogenic group isolated from stool specimens of patients with acute enteritis in Ghent, Belgium.

Bacteria species of the genus *Arcobacter* were first isolated from aborted bovine and porcine fetuses in 1977 (*2*). Based on similar phenotypic characteristics, they were originally classified as aerotolerant *Campylobacter* spp., until a separate genus was introduced in 1991 (*2*). Since then, 18 species have been identified and new species are pending. Members of the genus *Arcobacter* are aerotolerant gram-negative bacteria and able to grow at temperatures <30°C, which differentiate them from the *Campylobacter* species. The species *A. butzleri*, *A. cryaerophilus*, *A. skirrowii*, *A. cibarius*, *A. thereius*, and *A. trophiarum* have been identified in livestock worldwide and have been isolated from food of animal origin. Though these species have been associated with illness in farm animals, they are also known to colonize healthy animals (*3*).

*Arcobacter* have been classified as emergent pathogens by the International Commission on Microbial Specifications for Foods (*4*). Currently, 3 species have been reported to infect humans. Contaminated drinking water and raw or undercooked food that is eaten or handled are the most frequent sources of human infection (*5*,*6*). An association of *A. butzleri* and *A. cryaerophilus* with enteritis, colitis, and septicemia has been proposed in epidemiologic studies, outbreak reports, and case reports (*7*), although *A. cryaerophilus* can also be present in healthy humans (*8*). More recently, *A. skirrowii* has been implicated as the causal agent of bacteremia and colitis in individual case reports (*9*).

Information about transmission, colonization in the gut, and virulence in humans of members of the *Arcobacter *genus is insufficient. The presence of virulence genes and their distribution within the genus have been proven, but their role in pathogenicity in selected host groups has not yet been demonstrated (*10*).

Most laboratories do not use appropriate culture methods or conditions to detect species other than *Campylobacter jejuni* and *C. coli* from feces. Furthermore, identification of *Campylobacter* and related organisms to species level is not always performed or is executed with phenotypic methods, leading to conflicting results. Hence, data on the incidence and clinical importance of *Arcobacter* remain scarce. The aims of this study were to assess the feasibility of *Arcobacter* culture in a clinical microbiology laboratory and to determine the recent prevalence of *Arcobacter* spp. in humans with gastrointestinal disease in Belgium.

## The Study

Fecal samples were collected from outpatients and patients with symptoms of enteritis who were admitted for <72 hours to the Sint-Lucas Hospital in Ghent, Belgium, during 2008–2013. All age groups were represented. We examined the fecal samples macroscopically for consistency and presence of blood and mucus and microscopically for leukocytes and cultured them for all common bacterial pathogens. Feces of patients <2 years of age were also tested for rotavirus and enteric adenovirus, and those of adult patients with loose or mucous-containing stools were also tested for toxigenic *Clostridium difficile*. Samples were tested for parasites on clinical demand only.

We cultured for *Arcobacter* using an isolation method for fecal samples carried out in veterinary medicine that was previously validated for use on human feces (*11*): 1 g of feces was inoculated into a liquid selective broth (24 g/L *Arcobacter* broth with 50 mL lysed, defibrinated horse blood and an antimicrobial supplement of 16 mg/L cefoperazone, 10 mg/L amphotericin B, 100 mg/L 5-fluorouracil, 64 mg/L trimethoprim, and 32 mg/L novobiocine [Oxoid, Cambridge, United Kingdom]) and incubated for 24 h at 25°C in a microaerobic atmosphere of 6% O_2,_ 7% CO_2,_ 7% H_2_, and 80% N_2_ (Advanced Instruments, Inc., Norwood, MA, USA). Then, 40 μL of enriched broth was plated onto a solid *Arcobacter*-selective medium with the same composition as the broth but with the addition of 12 g/L agar technical no. 3 (Oxoid) and without the horse blood. The plates were incubated for 72 h at 25°C in a microaerobic atmosphere and examined daily. *Arcobacter* colonies were detected by screening the selective transparent agar as described with Henry transillumination microscopy for bluish colonies (*11*). Definitive identification was performed by a genus- and species-specific multiplex PCR, and subsequently, by amplification fragment length polymorphism (*12*,*13*).

Of the 8,994 eligible samples received during the study period, 6,774 (75.31%) samples were cultured for *Arcobacter*; 2,220 samples (24.68%) were excluded because of insufficient samples size. Of samples from the study population, *Campylobacter* spp. were isolated in 380 (5.61%), *Salmonella* spp. in 138 (2.04%), toxigenic *C. difficile* in 109 (1.61%), *Aeromonas* spp. in 16 (0.24%), and *Yersinia enterocolitica* in 13 (0.19%). Other gastrointestinal pathogens isolated in lower numbers included *Shigella* spp. (9 samples, 0.13%) and *Plesiomonas* spp. (3 samples, 0.05%).

*Arcobacter* spp. were isolated in samples from 89 patients (1.31%), ranking members of this genus as the fourth most commonly isolated pathogen group in the study. This ranking is comparable to those of other reports, although a higher isolation rate was obtained during this study (*14*,*15*). The distribution frequency of pathogens and of *A. butzleri* versus *A. cryaerophilus*, 49 (0.72%) and 38 (0.56%) isolates, respectively, is shown in Table 1. *A. skirrowii* was not recovered, but the first 2 known isolates of *A. thereius* (0.03%) from humans were documented. In the excluded population (n = 2,220), comparable recovery rates of *Campylobacter* spp. (6.53%), *Salmonella* spp. (2.88%) and *C. difficile* (2.07%) were observed. In 6 patients whose samples were positive for *Arcobacter*, a second pathogen was isolated: 1 patient was positive for *Salmonella* spp., samples from 2 patients showed *Campylobacter* spp. and *A. butzleri*, and toxigenic *C. difficile* was detected in 2 patients who tested positive for *A. butzleri* and in 1 patient who tested positive for *A. cryaerophilus*.

**Table 1:** Distribution of study population and number (%) of bacterial gastrointestinal pathogens during the study period 2008–2010 and 2012–2013, Belgium*

Characteristics	2008 (%)	2009 (%)	2010 (%)	2012 (%)	2013 (%)	5-y period (%)
Eligible samples	1,819	1,843	1,612	2,229	1,491	8,994
Included	1,375 (76)	1,374 (75)	1,112 (69)	1,768 (79)	1,145 (77)	6,774 (75)
Excluded	444 (24)	469 (25)	500 (31)	461 (21)	346 (23)	2,220 (25)
Pathogens identified						
Included patients						
* Campylobacter *spp.	64 (4.7)	54 (3.9)	68 (6.1)	85 (4.8)	109 (9.5)	380 (5.6)
* Salmonella* spp.	29 (2.1)	32 (2.3)	28 (2.5)	26 (1.5)	23 (2.0)	138 (2.0)
* Clostridium* *difficile*†	26 (1.9)	19 (1.4)	17 (1.5)	18 (1.0)	29 (2.5)	109 (1.6)
* Arcobacter* spp.	18 (1.3)	12 (0.9)	18 (1.6)	17 (0.9)	24 (2.1)	89 (1.3)
* Arcobacter butzleri*	6 (0.4)	7 (0.5)	11 (0.9)	7 (0.4)	18 (1.6)	49 (0.7)
* Arcobacter* *cryaerophilus*	12 (0.9)	5 (0.4)	5 (0.4)	10 (0.7)	6 (0.5)	38 (0.6)
* Arcobacter* *thereius*	0	0	2 (0.1)	0	0	2 (0.03)
* Aeromonas* spp.	2 (0.1)	3 (0.2)	6 (0.5)	1 (0.1)	4 (0.3)	16 (0.2)
* Yersinia enterocolitica*	1 (0.1)	4 (0.3)	1 (0.1)	2 (0.1)	5 (0.4)	13 (0.2)
* Shigella* spp.	2 (0.1)	2 (0.1)	3 (0.3)	2 (0.1)	0	9 (0.1)
* Plesiomonas* spp	0	2 (0.1)	0	0	1 (0.1)	3 (0.04)
Excluded patients						
* Campylobacter *spp.	20 (4.5)	23 (4.9)	39 (7.8)	27 (5.9)	36 (10.4)	145 (6.5)
* Salmonella *spp.	20 (4.5)	14 (3.0)	15 (3.0)	11 (2.4)	4 (1.2)	64 (2.9)
* Clostridium difficile*†	8 (1.8)	16 (3.4)	7 (1.4)	8 (1.7)	7 (2.0)	46 (2.1)

*The study was interrupted for 1 y in 2011. †Toxigenic strains only.

We collected retrospective data about age, hospital stay, presence of diarrhea, clinical diagnosis of enteritis, colonoscopy results, and underlying disease for the positive-culture patient group. We assessed differences between study populations and results using *t-*test and Χ^2^ statistical methods as appropriate.

The mean age of the included population (29 years) differed significantly (p<0.01) from the excluded group (22 years), because of an excess of infants from whom sample material was insufficient in the latter group. The positive-culture patient group had a mean age of 42 years and demonstrated a significantly older age distribution pattern (p<0.01) (Figure). The consistency of fecal samples was not predictive (p = 0.62) for the presence of *Arcobacter*. Within the *Arcobacter*-positive group, the mean age of patients whose specimens shed *A. butzleri* (49 years) was significantly higher (15 years, p<0.01) than that of patients whose specimens shed *A. cryaerophilus* (34 years). Presence of *A. butzleri* in patients admitted to the hospital and in outpatients was not significantly linked with underlying disease (p = 0.12). *A. cryaerophilus* was more frequently observed in outpatients who had uncomplicated gastroenteritis than in hospitalized patients with coexisting conditions (p<0.001) (Table 2).

**Figure:**
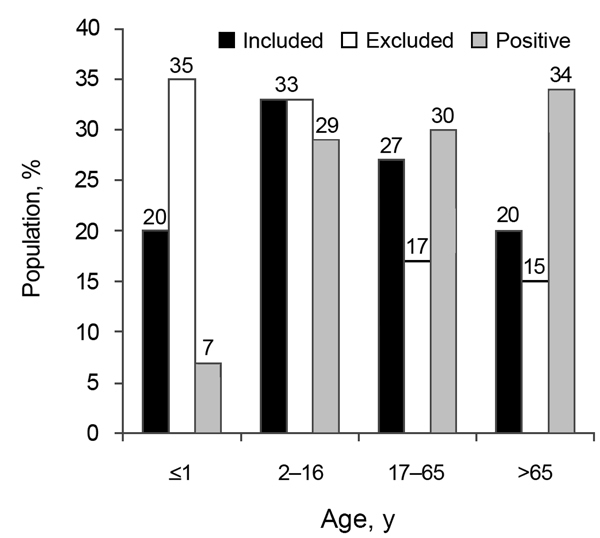
Age distribution of study population for detection of *Arcobacter* spp. in patients with acute enteritis, 2008–2013, Belgium. Black bars indicate percentage of age group included, white bars indicate percentage of patients excluded from the study, and dark gray bars indicate percentage of patients whose samples tested positive for *Arcobacter* spp.

**Table 2:** Microbiological and clinical details of 86 patients whose fecal samples contained *Arcobacter* spp., 2008–2013, Belgium

Characteristic	*Arcobacter* spp. samples, N = 89		
	*A. butzleri*, n = 49* (55%)	*A. cryaerophilus*, n = 38 (43%)	*A. thereius*, n = 2 (2%)
	No. (%)	No. (%)	No. (%)
Fecal consistency			
Solid	18 (37)	16 (42)	0
Semisolid-liquid	25 (51)	22 (58)	1 (1)
Mucous	5 (10)	0	1 (1)
Bloody	1 (2)	0	0
Clinical status			
Ambulatory	19 (39)	32 (84)	1 (1)
Hospitalized	30 (61)	6 (16)	1 (1)
Clinical syndromes			
Acute gastroenteritis	19 (39)	30 (79)	1 (1)
Coexisting medical condition	30 (61)	8 (21)	1 (1)
Chronic colitis	8 (15)	2 (5)	1 (1)

*49 samples from 46 patients.

## Conclusions

*Arcobacter* species were the fourth most common pathogen group isolated from fecal samples from persons with acute enteric disease. The high isolation rate could possibly be explained by the inclusion of an outpatient population, local indications for sampling, and the use of an enrichment culture method. The *Arcobacter*-positive patients tended to belong to older age groups. No notable association of *A. butzleri* enteritis with coexisting conditions was observed. The feasibility of selective culturing *Arcobacter* from fecal material in a routine microbiology hospital laboratory was confirmed, but the slow turnaround times for culture results show a need for optimization of methods. *Arcobacter* species should be considered and tested for in cases of diarrheal disease.
